# Novel Cellulose-Binding-Domain Protein in *Phytophthora* Is Cell Wall Localized

**DOI:** 10.1371/journal.pone.0023555

**Published:** 2011-08-24

**Authors:** Richard W. Jones, Manuel Ospina-Giraldo

**Affiliations:** 1 Genetic Improvement of Fruit and Vegetables Laboratory, United States Department of Agriculture-Agricultural Research Service, Beltsville, Maryland, United States of America; 2 Deptartment of Biology, Lafayette College, Easton, Pennsylvania, United States of America; University of Wisconsin-Milwaukee, United States of America

## Abstract

Cellulose binding domains (CBD) in the carbohydrate binding module family 1 (CBM1) are structurally conserved regions generally linked to catalytic regions of cellulolytic enzymes. While widespread amongst saprophytic fungi that subsist on plant cell wall polysaccharides, they are absent amongst most plant pathogenic fungal cellulases. A genome wide survey for CBM1 was performed on the highly destructive plant pathogen *Phytophthora infestans*, a fungal-like Stramenopile, to determine if it harbored cellulolytic enzymes with CBM1. Only five genes were found to encode CBM1, and none were associated with catalytic domains. Surveys of other genomes indicated that the CBM1-containing proteins, lacking other domains, represent a unique group of proteins largely confined to the Stramenopiles. Immunolocalization of one of these proteins, CBD1, indicated that it is embedded in the hyphal cell wall. Proteins with CBM1 domains can have plant host elicitor activity, but tests with *Agrobacterium*-mediated *in planta* expression and synthetic peptide infiltration failed to identify plant hypersensitive elicitation with CBD1. A structural basis for differential elicitor activity is proposed.

## Introduction

Cellulose binding domains (CBD), are highly conserved regions of family 1 carbohydrate binding modules (CBM), are generally associated with catalytic glycoside hydrolases, whose members include endoglucanases, exocellobiohydrolases and beta-glucosidases. The non-catalytic CBD aids in anchoring to polysaccharides, and is often separated from the catalytic region by a short, flexible linker region rich in serine, proline and threonine [1]. These carbohydrate binding modules aid in specific binding, but are required principally for binding to crystalline cellulose [2]. While CBM1 is ubiquitous on fungal saprophyte-encoded glycoside hydrolases, they have thus far been absent in most fungal plant pathogen-encoded glycoside hydrolases identified subsequent to the first fungal phytopathogen endoglucanase sequence reported [3]–[4]. Recently, genome sequence information has become available for the major plant pathogenic organism *Phytophthora* [5]–[6]. While sharing some similarities to phytopathogenic fungi, *Phytophthora* is classified within the Stramenopiles, separate from the fungal kingdom. In an effort to discover if *Phytophthora*-encoded glycoside hydrolases follow the structural paradigm of those found in fungal phytopathogens, a genome-wide survey for CBM1 in *Phytophthora infestans* was initiated. A total of five putative gene products were identified, however, none were associated with any form of catalytic domain. The gene products represent a novel group of *Phytophthora* proteins, with one or two cellulose binding domains. As *Phytophthora* cell walls are comprised largely of cellulosic glucans [7] it is probable that the cellulose binding proteins are associated with the cell wall. One *Phytophthora* glycoprotein with cellulose binding domains and a lectin binding region, referred to as CBEL, was found associated with the cell wall [8]. The CBEL protein, one of the cellulose binding domain proteins also identified in our search, elicits plant defenses [8]–[9]. In our study we have focused on a previously unrecognized, 13 kD cellulose binding protein, determining cellular location and elicitor activity.

## Results

**Figure 1:**
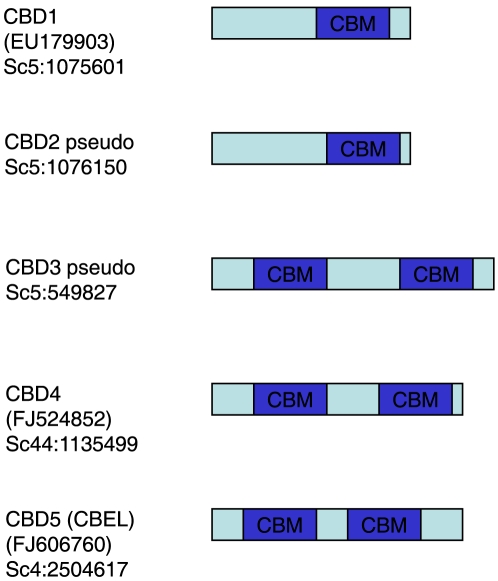
Identification of *Phytophthora infestans* genomic regions encoding the cellulose binding domain (Carbohydrate Binding Module 1) motif. The CBD2 and CBD3 gene regions have no corresponding ESTs.

**Figure 2:**
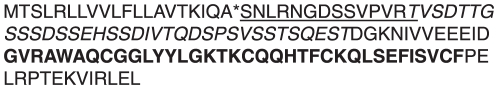
Sequence of a small cellulose binding domain protein (CBD1) encoded by *Phytophthora infestans*. Signal peptide cleavage site denoted by asterisk, underlined region represents peptide sequence used for antibody production, italicized letters represent predicted O-glycosylation region, and bold letters represent the conserved CBM1 sequence.

**Figure 3:**
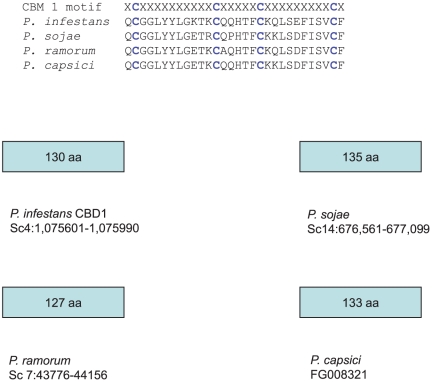
Identification of a small CBM1 protein in *Phytophthora* species. Disulfide bonds occur between C8:C25 and C19:C35.

We performed a genome-wide search of *Phytophthora infestans* genes encoding family 1 carbohydrate binding modules (CBM1) that are commonly found on cellulolytic enzymes from saprophytic fungi. There were very few CBM1 motifs detected (Figure 1), and none were associated with proteins having any type of catalytic domain. Analysis of corresponding EST data from the numerous *P. infestans* cDNA libraries that have been sequenced indicates that CBD1, CBD4 and CBD5 are transcribed. Our research focused on the previously undocumented CBD1, that is the smallest CBM1 containing protein (13 kD). The protein contains one CBM1, as opposed to CBD4 and CBD5 (corresponding to a protein known as CBEL), that contain two CBM1 regions. The protein has a signal peptide (www.cbs.dtu.dk/services/SignalP), a region with high probability of O-glycosylation (www.cbs.dtu.dk/services/NetOGlyc) and a CBM1 that is located near the C-terminus (Figure 2). Interestingly, the CBM1 ends about 14 amino acids from the terminus of a non-enzymatic protein, while CBM1 is situated at the extreme terminus of cellulolytic enzymes. Homologues of CBD1 are found in *P. sojae*, *P. ramorum* and *P. capsici* (Figure 3).

To test the ability of *P. infestans* CBD1 to elicit a plant response, expression in plants was mediated through infiltration of tobacco and potato with *Agrobacterium* carrying pBI121-cbd1. Six days after infiltration there were no signs of necrosis due to hypersensitive reaction to CBD1. Western blots showed that the protein was expressed *in planta* (data not shown). The possibility existed that the CBD1 protein was somehow sequestered and unable to interact with a potential receptor region, but this is unlikely as *in planta* expression of the *Phytophthora* CBEL protein elicited necrosis [9]. Synthetic peptides were also tested to determine if host defense could be elicited. The peptide, spanning the conserved CBM1 domain, was infiltrated at levels beyond that expected as biologically relevant, yet necrosis was not observed during a one week observation period.

**Figure 4:**
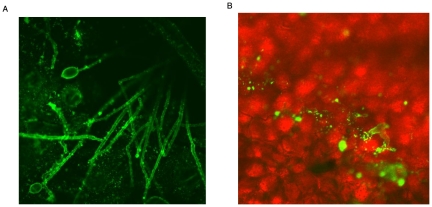
Immunofluorescent detection of hyphae and sporangia of *P. infestans*. Primary antibodies were prepared to a peptide representing the amino terminus (after signal peptide cleavage) of CBD1. Samples were viewed by confocal microscopy after incubation with FITC labeled secondary antibodies. A) hyphae and sporangia emerging from infected potato leaf tissue B) hyphae along the transition zone between symptomatic (upper right) and asymptomatic (lower left) infected leaf tissue.

During the initial isolation of CBD1, we precipitated proteins found in the culture medium. Using an anti-CBD1 antibody, we did not detect the protein in the filtrate. The antibody was then used for immunodetection, to determine if the protein was associated with the hyphae. A strong signal was apparent along the hyphal and sporangial walls of *P. infestans* that emerged from infected samples of potato leaf tissue (Figure 4A). The hyphae growing through the tissue was also labeled (Figure 4B).

To determine the association of CBD1 with the hyphal wall, various extraction methods were performed. Two effective methods were found, boiling in SDS or overnight incubation in Tris buffer (pH 9.5). Methods that would commonly elute CBM1 from cellulose were not effective [2], [ 18], [20]. The higher pH of 30 mM NaOH (11.5) was also not suitable. SDS extraction suggests interaction with other cell wall proteins. Total extractable CBD1 was quite low indicating a resilient association with the cell wall (data not shown).

## Discussion

This genome wide screen provides the first comprehensive evidence that *Phytophthora* retains the structural paradigm first found for phytopathogenic fungi [3]–[4], where cellulolytic enzymes are devoid of CBM1. Previous studies have clearly shown that *Phytophthora* encodes cellulolytic enzymes, such as family 5 and family 12 endoglucanases [10]–[11]. They are present as multiple copy gene families and there was no evidence of CBM1 motifs in these proteins.

The lack of CBM1 motifs on enzymes that may be critical to early penetration and invasion by biotrophic and hemibiotrophic fungi is becoming firmly established with the release of a wide range of genome sequences. Scanning numerous phytopathogenic fungal genomes we find that most lack CBM1 altogether. In special cases CBM1 can be identified with cellulolytic enzymes from phytopathogenic fungi, however they are found in necrotrophic pathogens. For example, *Pyrenophora tritici-repentis*, causal agent of tan spot disease of wheat, harbors a glycosyl hydrolase family 61 with a C-terminal CBM1 (accession XP_001936606). This particular fungus has a number of cellulolytic enzymes with CBM 1. Putative cellulolytic proteins with CBM 1 can also be detected in necrotrophic fungi such as *Phaeosphaeria nodorum* (accession EAT77212) and *Verticillium dahliae* (accession BQ109945).

Cellulolytic enzymes are not the only catalytic proteins that may contain a CBM1 region. In one very well documented case [12], the phytopathogenic fungus *Colletotrichum gloeosporiodes* f. sp. *malvae* was found to express two different pectin lyase genes, one included a CBM1 (accession AF158256). This is of particular interest as this phytopathogen undergoes a transition from hemibiotroph to necrotroph during disease progression. The pectin lyase with a CBM1 was only expressed during the necrotrophic phase.

The apparent negative selection against the presence of proteins with CBM1 motifs in phytopathogenic fungi is most likely due to their elicitor activity when recognized by potential host plants [13]. Plants do not encode CBM1 motifs, thus recognition and response to these motifs would be expected to limit the invasive ability of a fungus. Recent studies on CBEL (Carbohydrate-Binding Elicitor Lectin), a glycoprotein with two CBM1 regions, that is found in numerous *Phytophthora* species and is included in this genome survey, further supports the idea that CBM1 can act as a type of elicitor form referred to as a “pathogen-associated molecular pattern” or PAMP [8]–[9].

**Figure 5:**
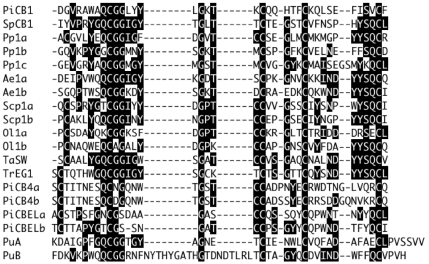
Comparison of a selection of cellulose binding domain (Carbohydrate Binding Module 1) proteins by alignment of conserved regions. PiCB1, *Phytophthora infestans* (CBD1) ABW76417; SpCB1, *Saprolegnia parasitica* AAZ94039; Pp1a, Pp1b and Pp1c, *Porphyra purpurea* AAA61792; Ae1a and Ae1b, *Aphanomyces euteiches* Ae_11AL5933; Scp1a and Scp1b, *Schizosaccharomyces pombe* NP_593986; Ol1a and Ol1b, *Ostreococcus lucimarinus* ABO94230; TaSW, *Trichoderma asperellum* ACB05430; TrEG1, *Trichoderma reesei* AAA34212; PiCB4a and PiCB4b, *Phytophthora infestans* (CBD4) ACL80756; PiCBELa and PiCBELb, *Phytophthora infestans* (CBD5) ACM68430; PuA and PuB, *Pythium ultimum* (CBD4) ADOS01001693. Arrow indicates previously reported site of mutation of tyrosine residue (ref. 17), found on the outer, non-cellulose binding face of the CBM1 of TrEG1. Designations of a, b and c refer to repeated CBM motifs within the same protein.

While the lack of elicitation by CBD1 or the synthetic peptide seems contrary to our initial hypothesis that CBM1 is recognized by plants [13], the basis for elicitor activity may reside in the structural subtleties of various CBM1 domains (Figure 5). A mutational analysis of *Phytophthora parasitica* var. *nicotianae* CBEL indicated that the tyrosine (Y 52 and Y 188) in each CBM1 was essential for elicitor activity. This tyrosine is conserved in the *Trichoderma* elicitors endoglucanase 1 (EG1) [13] and swollenin (SW) [14]–[15] as well as *P. infestans* CBEL. It is also present in *P. infestans* CBD4, which carries two CBM1 regions and, as with the other *Phytophthora* proteins containing a CBM1, has no apparent catalytic regions. A homologue of *P. infestans* CBD4 is found in *P. sojae* (accession AY183419) and was shown to elicit necrosis when expressed *in planta* through a Potato Virus X expression vector [16]. The tyrosine is also present in other CBM1 domains that haven't yet been tested for elicitor activity. The specific tyrosine, whose substitution eliminates elicitor activity, is absent in CBD1 from the four species of *Phytophthora* we have reviewed (Figure 3). Earlier mutational analysis of the CBM1 region from *Trichoderma* demonstrated that the same tyrosine (referred to as Y32 within the conserved CBM1) affects side chain conformations on the “rough” outer face of the CBM1 [17]–[19]. The inner “smooth” face of CBM 1 is involved in cellulose binding, while the outer face does not interact directly with cellulose. Therefore, while not essential for cellulose binding, this tyrosine plays a role in the three dimensional structure of the CBM1, suggesting that a specific structure must be retained for plant recognition. This further suggests that the cellulose binding activity, *per se*, may not be important to plant responses.

It should be noted that while CBM1 can act as an elicitor of plant defenses, the CBM1 needs to be exposed to allow for interaction with a yet undetermined plant molecule. The CBM1 on extracellular, cellulolytic enzymes is fully exposed after secretion and movement away from the microbial source, while the *Phytophthora* CBM1 containing proteins are wall bound, with no specific evidence that the CBM1 region is protruding or otherwise exposed. While a null response to a *Phytophthora* CBM1 would indicate that it was not an elicitor, a *positive* elicitation by introduction of *Phytophthora* CBM1 proteins as peptides, recombinant proteins or vectored recombinant proteins fail to represent the wall bound nature of these proteins. Therefore *Phytophthora* may be a successful biotroph during initial host infection by “hiding” the potential elicitor-positive CBM1 domains found in CBD4 and CBD5 in the hyphal wall. The CBD1 protein has a modified CBM1 domain that is “elicitor-negative”, allowing for localization on the surface of the hyphae.

The *P. infestans* CBD1 protein contains a signal peptide and a region with high probability of O-glycosylation, features suggesting extracellular localization. Proteins with O-glycosylation have been found associated with cell walls although it is unresolved as to whether Phytophthora has the machinery for O-glycosylation [20]. Interestingly, the three CBM1 proteins in *Phytophthora infestans* that are expressed; CBD1, CBD4 and CBD5 (CBEL), have *Phytophthora* homologues that have been associated with the cell wall [this study], [ 8, 20,].

Other proteins containing CBM regions have been associated with cell walls. One protein from *Schizosaccharomyces pombe* (accession NP 593986) contains two CBM1 regions and a GPI anchor, suggesting involvement at the cell membrane:wall interface. Another CBM-containing protein involved in synthesis of the cellulosic stalk of *Dictyostelium discoideum* (accession EAL 61319) is similar in size to *P. infestans* CBD1, however it contains a CBM49 [21]–[22]. The Stramenopiles *Aphanomyces* (www.polebio.scsv.ups-tlse.fr/aphano/) and *Saprolegnia* have numerous proteins with one or more CBM1 regions [23]–[24] that will likely be found associated with their cell walls. A single protein with two CBM1 regions was found in *Pythium ultimum*, while none were found in a search of *Hyalanoperonospora aradopsidis*. Notable is the fact that these proteins do not have a catalytic domain, thus the principal purpose of CBM1 proteins in the Stramenopiles seems to be in cell wall structure. In two organisms with distance relationship to the Stramenopiles [5]–[6], one can find the CBM1 domain. The brown alga *Porphyra* has a protein with three distinct CBM1 domains [25] and the diatom, *Ostreococcus lucimarinus* has a protein with two CBM1 modules and a leucine-rich repeat region (accession ABO94230). The additional CBM modules can provide much higher affinity for cellulose [26].

The presence of CBM1 may allow for interaction with cellulose outside of the cell wall. One protein, CBEL, was associated with adhesion to cellulosic substrates, however this protein harbors a lectin-like region that may also contribute to adhesion [27]. In the case of CBD4, as well as for CBEL, there are two CBM1 regions, which may allow for tethering of two separate glucan molecules. It will be interesting to determine where the CBM1 is situated relative to the rest of the protein, and if the CBM1 is embedded or exposed on the hyphal surface.

In addition to a possible role in cell wall synthesis, these proteins may play a role in hyphal permeability. While many fungi can survive in relative dry conditions, *Phytophthora* is referred to as a “water mold” and thrives under very moist and even aquatic conditions. The water mold fungi lack the wall associated hydrophobins present in other fungi that aid in desiccation survival. They are also more permeable to various compounds, including antibiotics [28]. The CBM1 proteins could play a role in regulating porosity of the hyphae. Another possible function is hyphal protection from enzymatic attack. The *Cladosporium fulvum* AVR4 protein has been shown to be a chitin binding protein that protects the hyphae from host chitinases during plant infection by binding to the surface of the hyphal wall [29]–[30]. The CBM1 proteins may help protect the overall integrity of the cell wall during attack by plant glucanases, however does not seem to be secreted outside the hyphal wall like AVR4. These proteins may also assist in protecting the cell wall from glucanases produces by saprophytic fungi that attack *Phytophthora* [31].

## Materials and Methods

### Culture conditions and inoculations

Liquid cultures of *Phytophthora infestans* were incubated at 20°C for three weeks in pea broth as previously described [10]. Infected plant tissue was prepared by inoculating detached leaflets of potato (cv. Green Mountain) with sporangia harvested from solid medium cultures as previously described [10]. Leaflets were maintained on moist paper towels in enclosed glass dishes for 5 days. Samples were harvested from infected leaves in the region spanning symptomatic to asymptomatic tissues, and fixed in methanol until further use.

### Gene identification and cloning

The carbohydrate binding module (CBM) family 1 motif (www.cazy.org) was used for tBLASTn searches of the genome of *Phytophthora infestans* (www.broad.mit.edu/annotation/genome/phytophthora_infestans). Additional genes encoding conserved CBM1 domains [32] were identified from searches in GenBank (www.ncbi.nlm.nih.gov/index.html).

The two small CBM1 encoding genes were cloned from *P. infestans* total RNA, extracted according to manufacturer's protocols for the Illustra kit (GE Healthcare, Buckinghamshire,UK). A cDNA pool was generated from total RNA using Superscript reverse transcriptase (Invitrogen, Carlsbad, CA, USA) and oligo dT reverse primer. DNA copies were generated from cDNA (100 ng) using forward primer *cb1f* (CGGTCCAAGCAGCACGCAGTCTCCG) and reverse primer *cb1r* (GGAATCGCTAGAGCTCCAGTCG). The PCR product was generated using Go Taq polymerase (Promega, Madison, WI, USA) with cycle parameters of 93 C for 3 min. followed by 35 cycles of 59 C for 30 sec., 72 C for 1 min., 93 C for 30 sec., and a final cycle of 72 C for 7 min. The product was cloned into TOPO TA cloning vector pCR-4 (Invitrogen) and transformed into *E. coli*. Plasmids were isolated from overnight shake cultures of transformed *E. coli* and sequenced.

### Antibodies and immunolocalization

The amino terminal portion of the processed protein was chosen as the antigenic target. The peptide sequence SNLRNGDSSVPVRT-C was synthesized and used to raise antibodies in rabbits (GenScript Corp., Piscataway, New Jersey, USA) with a final ELISA titer of 1∶6000.

Infected leaf tissue sections were rehydrated after methanol fixation by transfer to 50 mM Tris (pH 7.0). Samples were placed on concave microscope slides and incubated for 1 h at room temperature in Tris buffer (pH 7.0) plus 50 mM NaCl and 0.1% bovine serum albumin. Buffer was removed with an absorbent tissue and replaced with fresh buffer plus rabbit anti-CBD1 peptide (1∶300 dilution). Control samples were incubated with pre-immune serum. Samples were incubated for one hour, buffer removed and the sample rinsed three times for five minutes with fresh buffer. Secondary goat anti-rabbit, FITC labeled antibodies (Sigma, St. Louis, MO, USA) were added at a 1∶400 dilution and incubated for one hour. Samples were rinsed as previous and maintained in fresh buffer for confocal microscopy.

### Cell wall isolation and extraction

Harvested mycelial mats (3 g wt weight per sample) were rinsed twice by immersion in 30 ml of distilled water, and retained in 30 ml of distilled water on ice. Hyphae were comminuted for 1 min. with a Polytron homogenizer (Brinkmann, Westbury, NY, USA). Hyphal fragments were pelleted (3000 g, 10 min) followed by resuspension in 10 ml distilled water. The sample was sonicated for 20 sec. (Sonic Materials, Danbury, CT, USA ) followed by pelleting of hyphal fragments. The sample was washed with 40 ml distilled water and pelleted, subjected to a second round of sonication, washing and pelleting. The final samples, comprised of purified hyphal walls, were then used for extraction of wall-bound protein.

Each final sample (2.5 g wt weight) was extracted by individual extraction methods. Alkaline extraction was performed overnight at 4 C in 15 ml of 30 mM NaOH (pH 11.5), or 0.1 M Tris base (pH 9.5). Cellulose binding domain elution methods were performed overnight at 4 C in 15 ml using 10% polyethylene glycol or 0.25 M NaCl. Samples were also boiled for 5 min in 15 ml of 10% sodium dodecyl sulfate.

Samples from each treatment were pelleted (3000 g, 10 min) and the supernatant (10 ml) transferred to clean 50 ml polypropylene tubes. Ice-cold acetone (30 ml) was added to each supernatant, and tubes placed in a freezer (−20 C) overnight. Samples were centrifuged and the pellet was air dried.

Samples were resuspended in 0.2 ml Tris (pH 6.0), and 25 microliters added to an equal volume of Laemmeli buffer and placed in boiling water for 5 min. Samples were briefly centrifuged and loaded onto 4–20% gradient acrylamide gels (Life Therapeutics, Frenchs Forest NSW, Australia). Gels were run for three hours at 75 V, followed by electroblotting (3 h at 75 V) to nitrocellulose. Western blots were blocked with TBS plus 0.1% bovine serum albumin, incubated with anti-CBD1, rinsed and incubated with alkaline phosphatase labeled secondary antibody (Pierce, Rockford, IL, USA). After rinsing in Tris (pH 9.5) bands were detected by addition of Western Blue AP substrate (Promega).

### Expression in plant leaf by *Agrobacterium* infiltration

The TOPO-cbd1 plasmid (10 ng) was used with *Xba*1 containing forward primer *cbfa* (TCTAGACCTTTAGCCATAATGACC) and the *Sac*1 containing reverse primer *cbra* (GAGCTCTCACAACTCCAGTCGAATGAC). A PCR product was generated using Go Taq polymerase (Promega) and amplification performed with cycle parameters of 93 C for 2 min. followed by 35 cycles of ; 53 C for 30 sec., 72 C for 1 min., 93 C for 30 sec., and a final cycle of 72 C for 7 min. The product was cloned into TOPO TA cloning vector (Invitrogen, Carlsbad, CA, USA) and transformed into *E. coli*. Plasmid was isolated from overnight cultures of transformed *E. coli* and digested with *Xba*1 and *Sac*1 (Fermentas, Baltimore, MD, USA). The shuttle vector pBI121 (Invitrogen) was also digested with *Xba*1 and *Sac*1, releasing the beta-glucuronidase coding region. The restriction digested pBI121 was gel purified, along with the restriction digested CBD-encoding insert. The cbd insert was ligated into pBI121 and electroporated into *Agrobacterium tumefaciens* LBA4404 (Invitrogen). Cells were infiltrated with a syringe into the apoplast of leaves from tobacco (*Nicotiana tabacum*) and potato (*Solanum tuberosum*).

### Synthetic peptide infiltration

A synthetic peptide of 36 amino acids (GVRAWAQCGGLYYLGKTKCQQHTFCKQLSEFISVCF) spanning the conserved cellulose binding domain region (aa. 80–116) was synthesized (GenScript). This region encompasses the full CBM1. Peptide was dissolved in 50 mM sodium acetate pH 5.0 to a final concentration of 500 nM and infiltrated into the apoplast of leaves from tobacco and potato, using a needle-free syringe.
